# Effectiveness of vitamin D for adult patients with gingivitis

**DOI:** 10.1097/MD.0000000000018338

**Published:** 2020-01-10

**Authors:** Yao Feng, Dian-Song Yang, Hai-Bo Tang, Yuan-Sheng Ding, Xiao-Guang Li

**Affiliations:** a First Unit of Dental Pulp Disease Department; b Department of Radiation; c Department of Orthodontics, Second Affiliated Hospital of Jiamusi University, Jiamusi, China.

**Keywords:** effectiveness, gingivitis, safety, vitamin D

## Abstract

**Background:**

This study aims to explore the effectiveness of vitamin D for the management of adult patients with gingivitis.

**Methods:**

We will perform a comprehensive search from the following electronic databases: Cochrane Library, PUBMED, EMBASE, AMED, CINAHL, WANGFANG, VIP, CBM, and China National Knowledge Infrastructure. All databases will be searched from their inceptions to the present without language limitation. We will also search for unpublished data to avoid missing more potential studies. Two authors will carry out study selection, data extraction, and methodological quality evaluation, respectively. RevMan 5.3 software will be utilized for statistical analysis.

**Results:**

This study will summarize the up-to-date evidence about the anti-inflammatory effect of vitamin D for the management of adult patients with gingivitis through assessing modified gingival, gingival bleeding indices, inflammatory factors, plaque, quality of life, and any adverse events.

**Conclusion:**

This study may provide helpful evidence of vitamin D for the management of adult patients with gingivitis for clinical practice.

**Systematic review registration:**

PROSPERO CRD42019156561.

## 1 Introduction

Gingivitis is the mildest types of periodontal disease.^[1–3]^ If it is not treated fairly well, it will finally result in severe conditions or tooth loss in susceptible patients.^[4–6]^ It has been reported that its prevalence is about 55.7% among the adult population.^[7]^ It manifests as gums that are swollen, puffy, receding, and sometimes tender.^[8–10]^ Various risk factors are responsible for such disorder, including smoking, diabetes mellitus, and certain medications, such as oral contraceptives, genetic factors, and Vitamin D deficiency.^[11–18]^

Previous study has reported that vitamin D can be used as an alternative supplement for management of gingivitis.^[18–20]^ However, its results are still inconsistent and no systematic study has explored this topic. Therefore, this study will specifically and systematically assess the effectiveness of vitamin D for the management of adult patients with gingivitis.

## 2 Methods

### 2.1 Inclusion criteria

#### 2.1.1 Types of studies

We will include randomized controlled trials (RCTs) on assessing the effectiveness of vitamin D for the management of adult patients with gingivitis without language restrictions. However, we will exclude any other studies except RCTs.

#### 2.1.2 Types of patients

Any adult patients (more than 18 years old) who were diagnosed as gingivitis will be included without limitations of race and sex.

#### 2.1.3 Types of interventions

In the treatment group, all adult patients underwent vitamin D management.

In the control group, all adult participants received any other interventions, except vitamin D management.

#### 2.1.4 Type of outcome measurements

The primary outcome measures are modified gingival, as measured by the gingival-bleeding index or other indexes; and gingival bleeding indices, as assessed by whole-mouth mean bleeding index or other tools.

The secondary outcomes are inflammatory factors; plaque, as evaluated by whole-mouth mean plaque index or other scales; quality of life; and any adverse events.

### 2.2 Data sources and search strategy

#### 2.2.1 Electronic searches

We will carry out a comprehensive literature search in the following electronic databases: Cochrane Library, PUBMED, EMBASE, AMED, CINAHL, WANGFANG, VIP, CBM, and China National Knowledge Infrastructure. All electronic literature databases will be searched from their inceptions to the present without limitations of language and publication status. The sample search for Cochrane Library is shown in Table 1. We will also apply similar search strategy to other electronic databases.

**Table 1:**
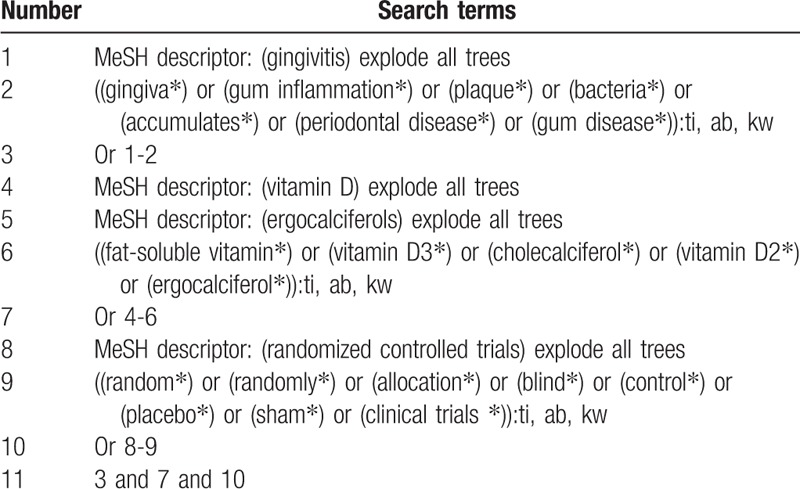
Search strategy for Cochrane Library.

#### 2.2.2 Other resources

In addition to the above electronic databases, we will also search for ongoing trials, dissertations, and reference lists of relevant reviews.

### 2.3 Data collection and analysis

#### 2.3.1 Study selection

We will use NoteExpress 3.0 to manage all searched records. First, we will classify and organize the preliminary searched studies and will exclude repeated and irrelevant studies. Second, 2 authors will scan the titles and abstracts of each literature according to the pre-designed eligibility criteria. After that, we will obtain full text of the remaining studies to identify their final eligibility for inclusion. Any inconsistent results between 2 authors will be solved by a third author through discussion. A flowchart that depicts the literature search process will be provided.

#### 2.3.2 Data extraction

Data from the included studies will be extracted by 2 independent authors using pre-tested data extraction sheet. The extracted information include study characteristics (such as author, journal, time of publication, etc), participants characteristics (such as sample size, age, sex, race, disease duration, diagnostic criteria, etc), study methods (such as methods of randomization, blind, etc), treatment details (such as types of intervention, dosage, etc), and outcomes (such as outcome results, adverse events, etc). Any differences will be solved through consultation with a third author. Any missing or unclear information will be contacted the primary authors to inquire them.

### 2.4 Risk of bias assessment

The risk of bias will be carried out by 2 authors according to the Cochrane risk of bias tool, respectively. It comprises of 7 specific aspects and each one is judged as low, unclear, or high risk of bias. Any disagreements will be managed to reach consensus with the help of a third author if necessary.

### 2.5 Subgroup analysis

We will conduct subgroup analysis to identify any possible reasons that may cause significant heterogeneity based on the different treatments, controls, and outcomes.

### 2.6 Sensitivity analysis

We will perform sensitivity analysis to check robustness of outcome results by excluding low-quality studies.

### 2.7 Reporting bias

We will use funnel plots to assess the reporting bias if more than 10 trials are included in this study.^[21]^

### 2.8 Statistical analysis

We will utilize RevMan 5.3 software to apply all statistical analysis. We will exert continuous data as mean difference or standardized mean difference and 95% confidence intervals (CIs), and dichotomous data as risk ratio and 95% CIs. Heterogeneity among included studies will be estimated using *I*^*2*^ statistic as follows: *I*^*2*^ ≤ 50% refers acceptable heterogeneity, while *I*^*2*^ > 50% exerts obvious heterogeneity. If acceptable heterogeneity is identified, a fixed-effects model will be used. Under such situation, we will plan to conduct meta-analysis if at least 2 eligible studies using the similar interventions, comparators, and outcome measurements are included. If obvious heterogeneity is found, a random-effects model will be applied, and a subgroup analysis will be performed. If we still identify obvious heterogeneity after subgroup analysis, we will just provide qualitative description for outcome results, instead of quantitative analysis.

### 2.9 Ethics and dissemination

This study will not need to provide research ethic, because this study will be based on the published data. We are expected to publish this study on peer-reviewed journals.

## 3 Discussion

To date, no appraisal for assessing the effectiveness of vitamin D for adult patients with gingivitis has been addressed. This is the first study to explore the effectiveness of vitamin D for the management of adult patients with gingivitis. In this study, we expect to appraise the effectiveness of vitamin D for adult patients with gingivitis. Besides, we will provide helpful recommendations on vitamin D for adult patients with gingivitis for either clinician or the future studies.

## Author contributions

**Conceptualization:** Yao Feng, Dian-Song Yang, Yuan-Sheng Ding, Xiao-Guang Li.

**Data curation:** Yao Feng, Hai-Bo Tang, Xiao-Guang Li.

**Formal analysis:** Dian-Song Yang, Hai-Bo Tang, Yuan-Sheng Ding.

**Funding acquisition:** Yao Feng.

**Investigation:** Yao Feng.

**Methodology:** Dian-Song Yang, Hai-Bo Tang, Yuan-Sheng Ding.

**Project administration:** Yao Feng, Xiao-Guang Li.

**Resources:** Dian-Song Yang, Hai-Bo Tang, Yuan-Sheng Ding, Xiao-Guang Li.

**Software:** Dian-Song Yang, Hai-Bo Tang, Yuan-Sheng Ding, Xiao-Guang Li.

**Supervision:** Yao Feng.

**Validation:** Yao Feng, Dian-Song Yang, Hai-Bo Tang, Yuan-Sheng Ding, Xiao-Guang Li.

**Visualization:** Yao Feng, Yuan-Sheng Ding, Xiao-Guang Li.

**Writing – original draft:** Yao Feng, Dian-Song Yang, Hai-Bo Tang, Yuan-Sheng Ding, Xiao-Guang Li.

**Writing – review and editing:** Yao Feng, Dian-Song Yang, Hai-Bo Tang, Yuan-Sheng Ding, Xiao-Guang Li.
